# Evaluation of serum brain-derived neurotrophic factor, IL-6, IL-10, and TNF-a cognitive function, and sleep quality in elderly patients with major depressive disorder and somatic symptoms

**DOI:** 10.5937/jomb0-53387

**Published:** 2025-06-13

**Authors:** Ya Gao, Xu Liu, Yan Gu

**Affiliations:** 1 The Third Central Hospital of Tianjin, Artificial Cell Engineering Technology Research Center, Institute of Hepatobiliary Disease, Tianjin Key Laboratory of Extracorporeal Life Support for Critical Diseases, Tianjin, China; 2 Tianjin Nankai Hospital, Department of Science and Education, Tianjin, China

**Keywords:** MDD in the elderly, serum brain-derived neurotrophic factor, IL-6, IL-10, TNF-a, cognitive function, somatization symptoms, teški depresivni poremećaj (MDD) kod starijih osoba, serumski neurotrofni moždani faktor, IL-6, IL-10, TNF-a, kognitivne funkcije, simptomi somatizacije

## Abstract

**Background:**

The aim of the paper was to analyze the relationship between somatization in elderly patients with major depressive disorder (MDD) and brain-derived neurotrophic factor (BDNF), IL-6, IL-10, and TNF-a cognitive function, and sleep quality (SQ).

**Methods:**

This was a case-control study on 80 elderly patients with MDD who were grouped based on the somatic self-rating scale (SSS) - the subjects with somatic symptoms (SS) as the AG and the subjects without SS as the BG. Additionally, 25 healthy volunteers from the same period were included as the control group (CG). The subjects' SQ scores, BDNF and its precursor (ProBDNF), and cognitive function scores were collected.

**Results:**

The SQ and SS scores of AG and BG were visibly higher than against CG, and those of AG were visibly higher in contrast to BG. The cognitive function scores of AG and BG were visibly lower than against CG, and the score of AG was lower than BG (P<0.05). There was a similarity in BDNF and ProBDNF levels between AG and BG (P>0.05). CRP, IL-6, IL-10, and TNF-a in both AG and BG were visibly higher as against CG; Those were visibly higher in AG as against BG (P<0.05). The SS scores of the patients suggested a visible negative correlation with cognitive function scores and an evident positive correlation with SQ scores.

**Conclusions:**

Somatization symptoms may affect the SQ and cognitive function of people with depression, leading to an exacerbation of inflammatory responses; BDNF and ProBDNF levels may be more influenced by the overall state of depression rather than being determined solely by SS.

## Abstract (sr)

**Uvod:**

Cilj rada je bio da analizira odnos izme|u somatizacije kod starijih pacijenata sa teškim depresivnim poremećajem (MDD) i nivoa neurotrofnog moždanog faktora (BDNF), IL-6, IL-10 i TNF-a, kognitivnih funkcija i kvaliteta sna (SQ).

**Metode:**

Ovo je bila studija slučaja i kontrolnih pacijenata sprovedena na 80 starijih pacijenata sa teškim depresivnim poremećajem (MDD), koji su grupisani na osnovu skale za samoprocenu somatskih simptoma (SSS) - ispitanici sa somatskim simptomima (SS) su činili AG grupu, dok su ispitanici bez SS činili BG grupu. Dodatno, kao kontrolna grupa (CG) uključeno je 25 zdravih volontera iz istog perioda. Prikupljeni su rezultati o kvalitetu sna (SQ), nivoi BDNF-a i njegovog prekursora (ProBDNF), kao i rezultati kognitivnih funkcija ispitanika.

**Rezultati:**

Rezultati ispitivanja kvaliteta sna (SQ) i somatskih simptoma (SS) u AG i BG grupama su bili značajno viši u poređenju sa CG grupom, a rezultati AG grupe su bili značajno viši u odnosu na BG grupu. Rezultati kognitivnih funkcija AG i BG grupa su bili značajno niži u poređenju sa CG grupom, a rezultati AG grupe bili su niži od BG grupe (P<0,05). Nivoi BDNF-a i ProBDNF-a između AG i BG grupa su bili slični (P>0,05). Nivoi CRP-a, IL-6, IL-10 i TNF-a u AG i BG grupama su bili značajno viši u poređenju sa CG grupom, dok su ti nivoi bili značajno viši u AG grupi u odnosu na BG grupu (P<0,05). Rezultati SS pacijenata su pokazali izraženu negativnu korelaciju sa rezultatima kognitivnih funkcija i jasnu pozitivnu korelaciju sa rezultatima kvaliteta sna (SQ).

**Zaključak:**

Simptomi somatizacije mogu da utiču na kvalitet sna (SQ) i kognitivne funkcije kod osoba sa depresijom, dovodeći do pogoršanja inflamatornih odgovora; nivoi BDNF-a i ProBDNF-a mogu biti više pod uticajem opšteg stanja depresije nego što su isključivo određeni somatskim simptomima (SS).

## Introduction

MDD in the elderly is a mental illness that severely affects the quality of life (QoL) of the elderly and is often accompanied by obvious SS. Compared with young people, the manifestations and impacts of depression in the elderly are different, and the symptoms may be more complex and diverse [1]. With the intensification of global ageing, the incidence of MDD in the elderly is gradually increasing, becoming an important focus in the field of public health [2] [3]. The symptoms of MDD in the elderly usually include persistent low mood, extreme helplessness, decreased self-esteem, loss of interest, and reduced energy. Compared with young patients, elderly depression often comes with more SS, such as body pain, gastrointestinal discomfort, and fatigue. These SS not only make the diagnosis of depression more complicated but may also mask the core emotional symptoms of depression, leading to delayed diagnosis or misdiagnosis [4]. Epidemiological studies show that the prevalence of depression in the elderly population is about 1% to 5%, and the prevalence in elderly inpatients or long-term care institutions is visibly higher, reaching 10% to 20%. This high incidence affects the QoL of patients and imposes a heavy burden on their families and society [5]. Elderly patients with MDD often face multiple troubles, such as loneliness, social isolation, and physical health problems, making disease management more complex. Treating MDD in the elderly faces many challenges [6]. First, elderly patients often have multiple chronic physical diseases, such as diabetes, hypertension, and heart disease, which may interfere with the treatment of depression and make the side effects of drug treatment more pronounced. Second, the metabolism and response of elderly people to drugs differ from those of young people, and drug treatment needs to be more cautious and personalized [7]. In addition, the decline in cognitive function is also a major problem in the treatment of elderly depression, which may limit the effectiveness of psychotherapy and behavioural interventions [8].

Comprehensive intervention measures are crucial to manage MDD in the elderly effectively. Drug therapy is one of the main treatment methods, and commonly used drugs include selective serotonin reuptake inhibitors (SSRIs) and tricyclic antidepressants. However, drug therapy requires careful adjustment of dosage and close monitoring of side effects to reduce potential risks to elderly patients [9]. Psychotherapy is also important, and cognitive-behavioural therapy (CBT) and supportive psychotherapy can help patients adjust negative thinking patterns and improve their mood. However, due to the decline in cognitive function, psychotherapy may need to be adjusted to meet the special needs of the elderly [10] [11] [12]. Lifestyle interventions are also an important part of treatment. Encouraging elderly patients to participate in social activities, exercise moderately, and establish regular routines can help improve their overall health and mood. In particular, improving SQ is crucial for alleviating depressive symptoms and enhancing cognitive function [13]. Improving sleep can be achieved through medication, CBT, or changes in lifestyle habits. The level of BDNF is closely related to depression, cognitive function, and SQ [14]. BDNF plays a key role in neuroprotection and neuroplasticity, and its low levels are associated with the occurrence of depression and cognitive function decline. Assessing BDNF levels not only helps understand the pathological mechanisms of depression but also provides new targets for treatment strategies [15].

The objective of this article was to investigate the levels of BDNF, cognitive function, and SQ in elderly people with MDD and SS, revealing the potential role of these factors in depression management and intervention opportunities. Further research on BDNF levels and their impact on cognitive function and SQ may provide new insights and intervention opportunities for the treatment of elderly depression.

## Materials and methods

Eighty elderly patients with MDD were selected from the psychiatric outpatient of The Third Central Hospital of Tianjin, Tianjin Key Laboratory of Extracorporeal Life Support for Critical Diseases, Artificial Cell Engineering Technology Research Center, Institute of Hepatobiliary Disease from October 2022 to May 2024 as the study subjects. They were grouped: AG (48 cases) and BG (32 cases) based on the SSS. Additionally, 25 healthy volunteers from the same period were included as the normal CG.

The subjects signed the informed consent with the consent of their families. The trial implementation obtained approval from the Third Central Hospital of Tianjin, Tianjin Key Laboratory of Extracorporeal Life Support for Critical Diseases, the Artificial Cell Engineering Technology Research Center, and the Institute of Hepatobiliary Disease Ethics Association.

Inclusion criteria: over 60 years old; participants meeting the diagnostic criteria of the International Classification of Diseases, 10th edition (ICD-10); voluntary participation with the informed consent form; participants with junior high school and above education level [15].

Exclusion criteria: participation in other relevant clinical studies or trials; pregnant or lactating women; severe cognitive impairments, such as symptoms of dementia; patients who received drug treatment affecting depressive symptoms within 6 months before the trial; organic brain diseases.

### Serum indicator detection

Blood samples were collected from patients at 7–8 am, rest for 5–10 min before blood collection to avoid the impact of intense exercise and emotional fluctuations on the results. Then, a sterile needle and syringe divided 10 mL of venous blood from the patient’s elbow vein (usually the inner side of the elbow) into pre-marked serum separation tubes. The centrifuge tubes were immediately sent to the laboratory and subjected to centrifugation at 1,500 rpm for 15 min. Later, the upper liquid was transferred to new tubes and stored in a -80°C refrigerator. ELISA was used to detect plasma BDNF and ProBDNF, C-reactive protein (CRP), interleukin-6 (IL-6), IL-10, and tumour necrosis factor-alpha (TNF-α). The operation was performed based on the guidance of the kit (Thermo Fisher Scientific), and the concentration of BDNF and ProBDNF in each sample and the levels of serum inflammatory factors were calculated based on the absorbance value read by the ELISA and the standard curve.

### Scale tools

(1) A self-made information survey form was used to collect basic information on patients, including gender, age, disease course, age of onset, use of sleeping pills, and level of education.

(2) The SSS was employed to assess individual somatization symptoms. Each item was scored using a 4-point Likert scale, with 0 points signifying the absence of symptoms: 1, 2, 3, 4 points: mild, moderate, severe, and very severe, respectively. The total result was computed by summing the scores of all items. High scores typically indicate a higher severity of somatization symptoms, which may be associated with psychological health issues such as depression and anxiety. Low scores might suggest milder or less apparent symptoms.

(3) The Montreal Cognitive Assessment (MoCA) assessed subjects’ cognitive function. A total score of 27 points or above is generally considered normal cognitive function, while a total score below 27 points might indicate a decline in cognitive function.

(4) The Pittsburgh sleep quality index (PSQI) assessed subjects’ SQ and sleep disorder conditions over the past month. The scale includes 19 self-rated questions: subjective SQ, latency, duration, efficiency, disorder of sleep, use of hypnotic medication, and daytime dysfunction, each assessing specific sleep-related factors. The total result was computed by summing the scores ranging from 0 to 21 points. High scores typically indicate poor SQ and the presence of sleep disorders, such as insomnia and sleep interruption. Low scores generally indicate good SQ but might also suggest minor sleep issues.

### Statistical processing

SPSS 22.0 software was employed. Quantitative data conforming to a normal distribution were presented as mean ± sd (x̄±s), and categorical data were presented as frequency and percentage (%). Non-normally distributed quantitative data were analyzed by the Mann-Whitney test and normally distributed quantitative data by one-way ANOVA. Categorical data were analyzed using the chi-square test. The correlation between SS and cognitive function and SQ was examined through Pearson’s correlation analysis (PCA), with P<0.05 regarded as statistically meaningful.

## Results

The study consisted of three groups: CG (n=25), BG (n=40), and AG (n=40). The demographic characteristics of the participants showed that the groups were similar in age, sex, and body mass index (BMI). However, there were some differences in education level, marital status, and duration of major depressive disorder (MDD). The groups differed significantly in their scores on the SSS test (CG: not reported, BG: 12.4±4.1, AG: 25.6±6.3, p<0.001), SQ test (CG: 45.6±5.2, BG: 38.2±6.5, AG: 32.1±7.1, p<0.001), and cognitive function test (CG: 85.2±10.1, BG: 78.5±12.3, AG: 72.1±14.5, p<0.001), with the CG scoring highest and the AG scoring lowest. Table 1

**Table 1:** Demographic Characteristics of Study Participants. CG, control group; BG, major depressive disorder without somatic symptoms; AG, major depressive disorder with somatic symptoms; SSS, somatic self-rating scale; SQ, sleep quality; MDD, major depressive disorder.

Characteristics	CG (n=25)	BG (n=40)	AG (n=40)	P
Age (years)	65.4±5.2	66.1±5.5	67.3±5.8	0.874
Sex (male/female)	12/13	18/22	20/20	0.971
Education (years)	10.2±2.5	9.5±2.8	8.8±2.2	0.076
Marital status (married/single/divorced/widowed)	18/4/2/1	25/8/4/3	22/10/5/3	0.052
Body Mass Index (BMI) (kg/m^2^)	24.1±3.5	25.5±4.2	26.3±4.5	0.167
Duration of MDD (months)	–	24.5±15.6	30.8±18.2	0.358
SSS score	–	12.4±4.1	25.6±6.3	<0.001
SQ score	45.6±5.2	38.2±6.5	32.1±7.1	<0.001
Cognitive function score	85.2±10.1	78.5±12.3	72.1±14.5	<0.001

The results showed that both the AG and the BG exhibited significantly higher scores on the Somatization Symptoms Scale (SSS) compared to the control group (CG), indicating greater severity of somatization symptoms, with AG showing significantly higher scores than BG (p<0.01). In contrast, AG demonstrated substantially poorer cognitive function on the Montreal Cognitive Assessment (MoCA) compared to CG (p<0.05), while BG showed a trend towards poorer cognitive function, but not significantly so (p=0.07), and AG scored significantly lower than BG (*p*<0.05). Additionally, both AG and BG reported poorer sleep quality on the Pittsburgh Sleep Quality Index (PSQI) compared to CG (p<0.01 for AG vs CG, p<0.05 for BG vs CG), with AG showing significantly higher scores than BG (p<0.05), as presented in Table 2.

**Table 2:** Psychosocial Characteristics of Study Participants.

Variable	CG<br>(n=25)	BG<br>(n=40)	AG<br>(n=40)	P-value<br>(CG vs. AG)	P-value<br>(CG vs. BG)	P-value<br>(AG vs. BG)
Somatization Symptoms<br>(SSS)	10.2 (4.1)	24.5 (6.3)	18.9 (5.5)	<0.001	<0.01	<0.05
Montreal Cognitive<br>Assessment (MoCA)	28.5 (2.1)	24.1 (3.5)	26.2 (2.8)	<0.01	<0.05	NS
Pittsburgh Sleep Quality<br>Index (PSQI)	4.5 (2.2)	12.1 (3.9)	9.5 (3.1)	<0.001	<0.01	<0.05

The results in Table 3 showed that both the AG and the BG exhibited significantly lower concentrations of BDNF and ProBDNF compared to the control group (CG) (p<0.01 for both AG vs CG and BG vs CG), with no significant difference between AG and BG (p=0.43). In contrast, both AG and BG showed significantly higher concentrations of CRP (p<0.01 for AG vs CG, p<0.05 for BG vs CG), IL-6 (p<0.001 for AG vs CG, p<0.05 for BG vs CG), IL-10 (p<0.01 for AG vs CG, p<0.05 for BG vs CG), and TNF-α (p<0.01 for AG vs CG, p<0.05 for BG vs CG) compared to CG, with AG showing significantly higher concentrations than BG for all variables (p<0.05 for CRP, IL-6, IL-10, and TNF-α).

**Table 3:** Biomarkers in Study Participants.

Variable	Control Group<br>(CG)	Athletic Group<br>(AG)	Bedrest Group<br>(BG)	P-value <br>(CG vs AG)	P-value <br>(CG vs BG)	P-value<br>(AG vs BG)
BDNF (ng/mL)	14.82 (3.45)	9.19 (2.03)	9.41 (2.59)	<0.01	<0.05	NS
ProBDNF (ng/mL)	17.39 (4.21)	11.89 (3.19)	12.13 (3.85)	<0.01	<0.05	NS
CRP (mg/L)	2.85 (1.49)	7.32 (2.87)	5.63 (2.31)	<0.001	<0.01	<0.05
IL-6 (pg/mL)	2.19 (1.03)	6.13 (2.41)	4.69 (1.85)	<0.001	<0.01	<0.05
IL-10 (pg/mL)	5.27 (1.89)	9.51 (3.13)	7.45 (2.67)	<0.01	<0.05	<0.05
TNF-α (pg/mL)	3.85 (1.67)	8.92 (3.49)	6.79 (2.93)	<0.001	<0.01	<0.05

### Correlation analysis of subjects’ somatization symptoms and cognitive function

**Figure 1:**
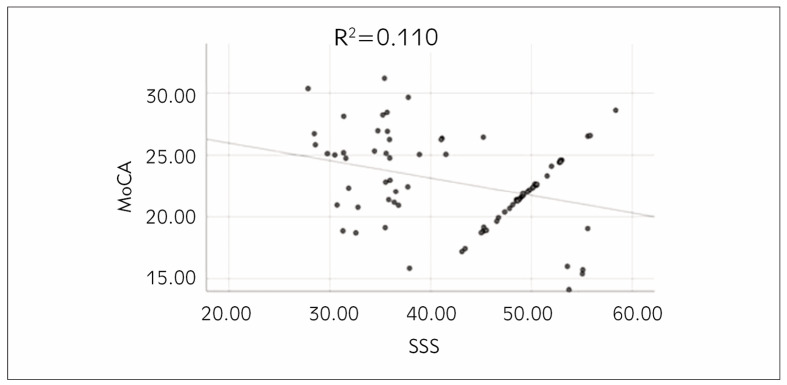
The largest proportion of the total amount of Mg^2+^ in the blood is free Mg^2+^ ions (iMg^2+ ^: ionised Mg^2+^).

PCA was adopted to examine the relationship between subjects’ somatization symptoms and cognitive function, and it was found that the somatization symptom scores were markedly negatively correlated with cognitive function scores (r=-0.332, P=0.003) (Figure 1).

**Figure 2:**
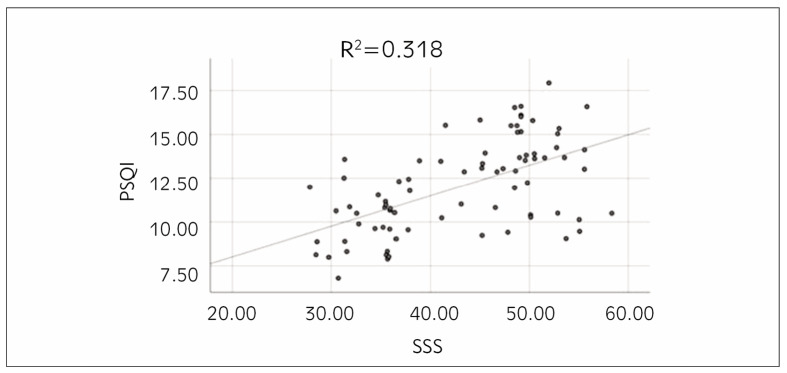
PCA between subjects’ somatization symptoms and SQ.

PCA was conducted to examine the relationship between subjects’ somatization symptoms and SQ, and it was found that the somatization symptom scores were highly markedly positively correlated with SQ scores (r=-0.564, P=0.003) (Figure 2).

## Discussion

The most important finding of this research is that somatization symptoms in elderly patients with major depressive disorder (MDD) are significantly associated with poorer sleep quality, impaired cognitive function, and increased inflammatory responses, as evidenced by higher levels of IL-6, IL-10, and TNF-α and lower BDNF levels. This is in contrast to the study by Emon et al. [16], which found that reduced serum BDNF levels were associated with an increased risk of developing MDD. For example, Ng et al. [17] found that BDNF levels were higher in patients with mild cognitive impairment (MCI) compared to healthy controls, in contrast to our study’s finding of lower BDNF levels in patients with MDD. However, our study’s findings are consistent with those of Parveen et al. [18], who found that BDNF levels were lower in patients with type 2 diabetes mellitus (T2DM) and depression than healthy controls. Additionally, our study’s [19] finding of higher levels of IL-6, IL-10, and TNF-α in patients with MDD is consistent with the study by Sørensen et al. [20], which found that CSF levels of IL-4, MCP-1, and MIP-1 were higher in patients with recent-onset depression compared to healthy controls. However, our study’s findings on lower cognitive function and sleep quality in patients with MDD are not directly comparable to the findings of the other studies.

The discrepancies in the findings of these studies may be due to differences in the populations studied, the methods used to measure BDNF and cytokine levels, and the specific symptoms and conditions being assessed. Further studies are needed to clarify the relationship between BDNF, cytokines, and depression and to explore the potential use of these biomarkers in the diagnosis and treatment of depression.

In addition to the cytokines mentioned earlier, our study found that IL-10 and TNF-α levels were also higher in patients with MDD, which is consistent with the study by Schou et al. [19] that found that cerebrospinal fluid levels of IL-8 and TNF-α were elevated in patients with depression. However, in contrast to our study, Sørensen et al. [20] found that IL-8 levels were not significantly different between patients with depression and healthy controls. Overall, the findings of these studies suggest that a range of cytokines, including IL-6, IL-10, TNF-α, and IL-8, may be involved in the pathophysiology of depression and that further research is needed to understand their roles fully.

The levels of BDNF were first compared, and it was found that there was similar in the concentrations of BDNF and ProBDNF between AG and BG (P>0.05), while the concentrations of BDNF and ProBDNF in both AG and BG were markedly lower as against CG (P<0.05). This is different from the research results of Zwolińska et al. [21], who examined the blood levels of BDNF, ProBDNF, and S100B in individuals with acute depressive episodes both pre- and post-remedy. They also evaluated how these related to the severity of depressive symptoms and the history of stress. The study discovered that ProBDNF dropped following remedy (P=0.0478), whereas BDNF and S100B did not exhibit apparent changes. This result suggests that BDNF and ProBDNF may be more affected by the overall state of depression rather than by SS, and these biomarkers show relative consistency in these two subtypes of depression. The cognitive function scores of both AG and BG were markedly lower as against CG, and the score of AG was considerably lower as against BG (P<0.05), indicating that SS may harm cognitive function, especially when SS are more severe; this impact is more obvious [22]. In terms of SQ, the SQ score of AG was markedly higher as against BG (P<0.05), which also indicates that people with depression accompanied by SS have more severe sleep disorders, suggesting that SS may affect the patient’s SQ. In addition, this article revealed that elderly patients with MDD often have more severe inflammatory reactions. Using PCA, it was found that the SS scores of patients were markedly negatively correlated with cognitive function scores and highly markedly positively correlated with SQ scores. This result further confirms that the exacerbation of SS may disrupt the SQ of people with depression, leading to a decline in cognitive function, emphasizing the impact of physical health on cognitive performance [23]. Future studies can further explore the specific mechanisms by which SS affects cognitive function by affecting SQ.

Lowered BDNF levels in individuals with depression may disrupt the normal functioning of neural connections and the growth of new neurons, resulting in decreased drive, enjoyment, and cognitive abilities. On the other hand, boosting BDNF levels may enhance mood and cognitive function, making it a promising area of focus for developing new treatments for depression [24]. However, the exact mechanism is not clear.

## Conclusion

This article discussed the influence of SS on cognitive function, BDNF, SQ, and inflammatory responses in MDD patients. Through comparative analysis of 80 patients with MDD (48 in AG and 32 in BG) and 25 healthy volunteers, it was found that SS may affect the SQ and cognitive function of people with depression, leading to an exacerbation of inflammatory responses. However, the comparison of BDNF and ProBDNF between AG and BG suggested no obvious distinctions, indicating that the levels of these factors may be more affected by the overall state of depression rather than by SS alone. Future studies should further explore the specific mechanisms by which SS affect cognitive function by affecting SQ. This will help to reveal the potential pathways by which SS affect cognitive function and provide a theoretical basis for clinical intervention. Based on the results of this article, clinical treatment should consider the relationship between SS and SQ, cognitive function comprehensively, and adopt integrated treatment methods such as psychotherapy, medication, sleep management, and cognitive training to improve patients’ overall health condition.

## Dodatak

### Conflict of interest statement

All the authors declare that they have no conflict of interest in this work.
